# Does the Academy of Breastfeeding Medicine’s Clinical Protocol #36 ‘The Mastitis Spectrum’ promote overtreatment and risk worsened outcomes for breastfeeding families? Commentary

**DOI:** 10.1186/s13006-023-00588-8

**Published:** 2023-09-05

**Authors:** Pamela Douglas

**Affiliations:** 1 The School of Nursing and Midwifery, Griffith University, Brisbane, Australia; 2 General Practice Clinical Unit, The University of Queensland, Brisbane, Australia; 3 Medical Director, The NDC Institute, ndcinstitute.com.au, Brisbane, Australia

**Keywords:** Lactation, Breastfeeding, Mastitis, Engorgement, Implementation science, Breast inflammation, Clinical protocol

## Abstract

**Background:**

In 2022 the Academy of Breastfeeding Medicine (ABM) published Clinical Protocol #36: The Mastitis Spectrum, which aims to update clinical approaches to management of benign lactation-related breast inflammation. The protocol has been timely because of the exponential increase in knowledge about the human milk microbiome over the past decade. This Commentary aims to continue respectful debate amongst clinicians and researchers within the Academy of Breastfeeding Medicine and more broadly, confident that we share a fundamental commitment to promote breastfeeding and support the well-being of lactating women, their infants and their families.

**Analysis:**

Although Clinical Protocol #36 offers advances, it does not fulfil the principles of best practice implementation science for translation of evidence into clinical guidelines. Clinical Protocol #36 inaccurately represents studies; misrepresents theoretical models as proven aetiologies; does not consistently attribute sources; does not reliably apply the SORT taxonomy; and relies upon single case reports. As a result, various recommendations in Clinical Protocol #36 lack an evidence-base or credible underlying theoretical model. This includes recommendations to use ‘lymphatic drainage’ massage, therapeutic ultrasound, and oral lecithin. Similarly, based on a contestable theoretical model which is presented as fact, Clinical Protocol #36 makes the recommendation to either reduce frequency of milk removal or to maintain current frequency of milk removal during an episode of breast inflammation. Although Clinical Protocol #36 limits this advice to cases of ‘hyperlactation’, the diagnosis ‘hyperlactation’ itself is undefinable. As a result, this recommendation may put breastfeeding women who present with breast inflammation at risk of worsened inflammation and decreased breast milk production.

**Conclusion:**

Clinical Protocol #36 offers some advances in the management of breast inflammation. However, Clinical Protocol #36 also exposes clinicians to two international trends in healthcare which undermine health system sustainability: overdiagnosis, including by over-definition, which increases risk of overtreatment; and antibiotic over-use, which worsens the crisis of global antimicrobial resistance. Clinical Protocol #36 also recommends unnecessary or ineffective interventions which may be accessed by affluent patients within advanced economies but are difficult to access for the global majority. The Academy of Breastfeeding Medicine may benefit from a review of processes for development of Clinical Protocols.

## Background

In 2022 the Academy of Breastfeeding Medicine (ABM) published Clinical Protocol #36: The Mastitis Spectrum, which aims to update clinical approaches to management of benign lactation-related breast inflammation [1]. A great deal of work is invested in the development of a Clinical Protocol. This Commentary acknowledges and respects the authors’ commitment to offering the best possible clinical care for breastfeeding pairs. However, this Commentary shares Baeza et al’s grave concerns about Clinical Protocol #36’s scientific integrity [2].

Clinical Protocol #36 introduces some concepts and recommendations that align with the analyses and the clinical guidelines for lactation-related breast inflammation published as part of the breastfeeding domain of Neuroprotective Developmental Care earlier that same year [3–6]. Specifically, elements that align include that breast inflammation:

- Is a spectrum condition.
- Is not helped by and is likely to be worsened by deep lump massage.
- May elicit a systemic response that is not necessarily infective.
- Mostly resolves with conservative care.
- Is not the same as normal lactational glandular tissue which can feel ‘lumpy’ and tender.
- Does not develop into infection in a period of hours.
- Is not caused by retrograde spread of bacteria from a damaged nipple.
- Is not caused by mammary candidiasis.
- Is not helped by.
  - instructions to have baby ‘drain’ the breast.
  - ‘dangle’ feeds.
  - topical applications.
- Does not require investigations of c-reactive protein or white blood cell count, since these are markers of inflammation not specific for infection.

Despite the above commonalities, I agree with Baeza et al’s concern that ABM Clinical Protocol #36 contains significant scientific flaws [2]. These flaws give rise to clinical recommendations which may be either of no benefit or which may worsen outcomes for breastfeeding pairs and their families.

### Clinical protocol #36 does not conform with principles of scientific best practice

Clinical lactation support remains a research frontier [7]. Breastfeeding families and the health professionals who support them deserve clinical guidelines developed from rigorous application of implementation science [8, 9], even in the context of relative paucity of research. Best practice implementation science in health care requires:

- Systematic or metanarrative review of existing and interdisciplinary research, interpreted through the lens of clinical experience, from which theoretical frames are developed;
- Translation of theoretical frames and existing evidence into education programs or clinical guidelines;
- Collation of iterative feedback from patients in the clinic and in pilot studies;
- Improvement of education programs or clinical guidelines in response to feedback;
- Layers of evaluative studies, qualitative and quantitative.

Clinical Protocol #36 fails to comply with best practice implementation science in the following ways:

1. Inaccurate representation of existing studies.
2. Theoretical models misrepresented as facts.
3. Sources not reliably attributed.
4. Strength of Recommendation Taxonomy (SORT) not reliably applied.
5. Reliance upon single case reports.

### Inaccurate representation of existing studies

To build theoretical frameworks translatable into effective recommendations, implementation science requires accurate representation and critical analysis of existing studies. Examples of studies inaccurately represented in Clinical Protocol #36 are detailed in Table 1.

**Table 1:** Analysis of accuracy of Clinical Protocol #36’s representation of research

Quote from Clinical Protocol #36	Analysis
“A Swedish study noted that most women with inflammatory mastitis had complete resolution of symptoms without need for antibiotics or other interventions. The authors attributed this finding to a focus on symptomatic control, appreciation of the physiological anti-inflammatory response, and regular communication between patient and clinician [Kvist 2007]” [1, p. 366].	In Kvist et al. 2007 participants with breast inflammation received intervention from midwife which included: • Unspecified fit and hold advice; • Advice which aimed to decrease intervals between feeds; • Advice about emptying the breast by manual expression; and • Advice about warm showers and pumping the breast [10].
“A systematic review concluded that although breast massage may reduce pain, it should not be recommended as standard of care because it requires extensive training to master atraumatic approach [Anderson et al 2019]. The most successful technique [Gua Sha] approximates manual lymphatic drainage with light sweeping of the skin rather than deep tissue massage [Witt et al 2016; Ezzo et al 2015]” [1, p. 368]	Anderson et al. conclude: “The overall effect of breast massage on reported outcomes is uncertain” [11, p. 1679]. • Gua Sha scrapes lightly from the base of the breast towards the nipple with a specialised soft instrument; patients reported decreased pain 5 and 30 min afterwards. This is opposite to light massage from nipple towards the axilla delivered in Manual Lymphatic Drainage and Therapeutic Breast Massage in Lactation (referred to as ‘lymphatic drainage’ in Clinical Protocol #36).
“Consider lymphatic drainage to alleviate interstitial edema [Ezzo et al 2015]. Figure 21” [1, p. 372] “Figure 21. Technique of lymphatic drainage” [1, p. 371]	Ezzo et al’s 2015 Cochrane review analysed studies which combined Manual Lymphatic Drainage (MLD) with compression bandaging for breast-cancer related lymphoedema in the upper limb after surgical axillary node dissection or radiation therapy; found no benefits for limb pain and heaviness of lymphedema; contradictory or inconclusive evidence concerning improved function and quality of life [12]. • Recommending ‘lymphatic drainage’ on the basis of Ezzo et al. conflates limbs after breast cancer surgery or radiotherapy with the radically different tissue environment of the lactating breast. • Systematic reviews of efficacy of MLD in 2020 and 2021 also show little benefit, suggesting prolonged tissue compression alone is the active ingredient [13–16]. • Witt et al. doesn’t demonstrate efficacy of ‘lymphatic drainage’ massage (also Therapeutic Breast Massage for Lactation TMBL) for breast inflammation [17], including because: o Component evaluating TBML for plugged ducts (n = 17) and mastitis (n = 7) is pre- and post-study of small numbers; o TBML intervention includes milk removal by infant or by hand expression, stimulating ductal dilations which explains possible efficacy [4]; o TBML delivered as part of a comprehensive breastfeeding intervention/consultation by IBCLC and/or breastfeeding medicine physician; o Component evaluating engorgement shows no improvement in pain at day 2 or week 12.
“Fig. 19. Ice and decreased removal of breast milk reduce ductal narrowing” [Zakarija-Grkovic & Stewart 2020] [1, p. 370] “Consider ice for symptomatic relief” [1, p. 370]	Cochrane review 2020 cold gel pack treatment for engorgement: • Uncertainty about effectiveness of cold gel packs on breast pain because very low certainty of evidence; • May be more effective than routine care for breast hardness in engorgement, but low-certainty evidence; • Little difference in women’s satisfaction compared to routine care [18]. In breast inflammation generally: • Warmth may increase stromal tension and duct compression by increasing blood flow; ductal dilation is not influenced by warmth, unless warmth is used as part of nipple stimulation. • Cold application decreases ductal diameters in the nipple, risking decreased milk transfer [19] [20]. • No evidence to support application of compresses, hot or cold.
“Sunflower or soy lecithin 5–10 g daily by mouth may be taken to reduce inflammation in ducts and emulsify milk [Mitchell & Johnson 2020; Chan et al 2003]” [1, p. 369]	No evidence cited to support efficacy and no plausible physiological mechanism. • In a methodologically weak retrospective audit by Mitchell & Johnson, 34 women were treated for nipple blebs, claimed to be caused by mammary dysbiosis, including with lecithin (all) and antibiotics (44%) [21]. • In Chan et al. 2003 study, lecithin directly added to a test tube of milk from mothers of prematurely born infants resulted in less loss of fat because the milk fats were less likely to adhere to the collecting device [22].
“Therapeutic ultrasound or TUS uses thermal energy to reduce inflammation and relieve edema. TUS may be an effective treatment for conditions arising in the mastitis spectrum [Mogenson et al 2020]” [1, p.370]	Mogenson et al. is narrative review of non-pharmacological approaches to pain, engorgement and plugging in lactation, not data which supports the use of TUS [23]. • Mechanisms by which TUS is proposed to “[use] thermal energy to reduce inflammation” not clarified. • Diepeveen et al. 2019 noted little empirical evidence to support the use of TUS in lactation-related breast inflammation despite common use by Australian physiotherapists [24]. • McLachlan et al. 1991 reported TUS no more effective than placebo for engorgement [25]. • A 2012 retrospective study of 25 mothers found that 23 had resolution of plugged duct following TUS but serious methodological weaknesses [26]. • Appropriate ultrasound frequency unknown; penetration depth investigated in non-breast tissue only.
“Consider probiotics” [Crepinsek et al 2020; Oikonomou et al 2020; Amir 2016; Barker et al 2020; Fernandez et al 2016; Hurtado & Fonolla 2018]” [1, p. 370]. Levels of evidence: 1–2. Strength of recommendation: B “Bacterial mastitis represents a progression … to an entity necessitating antibiotics or probiotics to resolve” [1, p. 363] “Probiotics have been shown not to alter composition of human milk microbiome [Crepinsek et al 2020; Oikonomou et al 2020; Amir 2016; Barker et al 2020]” [1, p. 372]	Citation of Hurtado & Fonollo is Letter to Editor, not a study; likely meant to be Hurtado et al. 2017 [27] • Barker et al. 2020 review identified 5 RCTs investigating probiotic consumption for treatment (3 studies) or prevention (2 studies) of mastitis, including Fernandez et al. 2016 [28] and Hurtado et al. 2017; noted significant methodological limitations concerning baseline characteristics, study hypotheses, lack of power calculations, definitional issues, potential conflicts of interest; concluded no reliable supporting evidence exists [29]. • Simpson et al. 2018 found no change in human milk microbiome composition when 415 breastfeeding women were randomized to receive probiotics or placebo [30].
“Avoid the use of nipple shields. Available evidence does not support the use of nipple shields. Neither safety nor effectiveness has been demonstrated. Nipple shields … result in inadequate breast milk extraction [McKenchie & Eglash 2010]” [1, p. 367]. Level of Evidence 3. Strength of recommendation C.	• A 2015 systematic review and 2021 review conclude that nipple shield use substantially benefits breastfeeding when problems emerge, in measurable outcomes and in reports by mothers [20, 31]. • A 2021 study randomized nipple shield use in 20 mothers with nipple pain compared to 28 without, finding nipple shields improved maternal comfort; did not impact milk removal or sucking strength in the pain group [32]. • Nipple shield use often masks failure to address underlying problems of positional instability or conditioned dialling up at the breast [6] but may be effective adjunct support for nipple pain and damage, concurrent with fit and hold repair.
“It should be noted that ultrasound studies documenting a small number of orifices approaching the nipple [Ramsay 2005] reflect limitations of radiographic images as compared with histological anatomy” [1, p. 363]	• Ramsay et al. showed dense glandular and duct tissue within a 3 cm radius of the base of nipple, and on average 9 main ducts (range 4–18) [33]. • Histological studies which reveal more nipple duct orifices than demonstrated in Ramsay et al’s ultrasound study not cited. • Purported limitations of Ramsay et al’s ultrasound studies relative to histology not clarified.

### Theoretical models misrepresented as fact

Given that clinical lactation support is a research frontier with a relative paucity of evaluative studies to guide clinicians, clinical advice often relies upon theoretical models. Theoretical models or hypotheses need to be explicit: named, described, and debated. Naming a theoretical model and clarifying its proposed pathophysiological mechanisms is important for scientific integrity and transparency. Misrepresenting theoretical models as fact in clinical guidelines misleads clinicians.

#### Example A. Clinical Protocol #36 asserts that dysbiosis is one of two fundamental causes of lactation-related breast inflammation

Clinical Protocol #36 states as fact that “under physiological conditions, coagulase-negative Staphylococci and viridans Streptococci (i.e. *S mitis* and *S salivarius*) form thin biofilms that line the epithelium of the mammary ducts, allowing a normal milk flow” [1, p. 365]. The physiological, cellular, or biochemical reasons why coagulase-negative *Staphylococcus* and *Streptococcus* bacteria – and why these genera and not other micro-organisms – might form a physiologic ductal biofilm (instead of remaining planktonic) in healthy lactating women are not discussed.

Clinical Protocol #36 continues: “In the setting of dysbiosis these species proliferate and function under opportunistic circumstances whereby they are able to form thick biofilms inside the ducts, inflaming the mammary epithelium” [1, p. 365]. The pathophysiological mechanisms by which ‘dysbiosis’ develops and turns physiological ductal biofilm into pathological biofilm are also not discussed. This pathogenic microbiota hypothesis of lactation-related breast inflammation is further presented as fact in Fig. 1 and schematically illustrated in Fig. 2 of Clinical Protocol #36. The latter illustration is adapted from Figure 1 in an article by Fernandez et al, published in 2014 prior to recent advances in human microbiome and human milk microbiome science [34, 35].

However, the latest human milk microbiome research renders the pathogenic microbiota hypothesis of lactation-related breast inflammation outdated [4]. ‘Eubiosis’ needs to be defined before ‘dysbiosis’ can be described. Taxonomic categorisation of ‘eubiosis’ in human microbiomes is increasingly considered unachievable and less relevant; the research focus has shifted to microbiome functionality, which emerges from complex interactions between various elements [36–43]. The human milk microbiome, leucocytes, epithelial cells, oligosaccharides, exosomes and metabolome are each complex systems interacting within the complex adaptive system of the mammary immune system [4]. Composition of the human milk microbiome is extremely variable both within the one woman over time, and between lactating women.

Kvist et al. found no correlation between scores for erythema, breast tension, pain or total severity of symptoms and the type of bacteria in breast milk during episodes of breast inflammation [44]. High counts of *Staphylococcus aureus* and decreased microbial diversity associated with breast inflammation are most plausibly explained as secondary to wound-healing inflammatory responses of the mammary immune system, rather than as *causes* of breast inflammation [4]. That is, the dramatic influx of leucocytes (with their powerful bactericidal properties) into alveoli and lactiferous duct lumens as a result of an inflammatory cascade is likely to decrease counts of more susceptible bacteria and increase counts of more adaptive bacteria like *S. aureus*, without typically tipping the patient into an infective process requiring antibiotic treatment [4].

Clinical Protocol #36 implicitly acknowledges the implausibility of the pathogenic biofilm hypothesis by classifying postpartum engorgement as “a distinct clinical entity related to interstitial edema and hyperemia” [1, p. 362]. This separate classification seems to acknowledge that engorgement is unlikely to result from generalised narrowing of lactiferous ducts by whole-of-breast pathological biofilm. Yet Clinical Protocol #36 doesn’t offer pathophysiological mechanisms to explain the interstitial fluid and hyperaemia of engorgement.

Applying the Neuroprotective Developmental Care classification system which arises from the mechanobiological model of lactation-related breast inflammation, engorgement belongs on the spectrum of breast inflammation, subject to the same aetiological model as other clinical presentations of benign lactation-related breast inflammation [4, 5].

#### Example B. Clinical Protocol #36 asserts that ‘hyperlactation’ is the second fundamental cause of breast inflammation

Clinical Protocol #36 states as fact that “ductal lumens can be narrowed by edema and hyperemia associated with hyperlactation”, not only by pathological biofilm formation [1, p. 361]. This implicitly acknowledges the mechanical effects of raised stromal pressure on lactiferous ducts, which are then compressed, as detailed earlier in the mechanobiological model of breast inflammation [4]. The causative role of ‘hyperlactation’ is further presented as fact in Fig. 1 of Clinical Protocol #36 [1].

Clinical Protocol #36 states Clinical Protocol #32 on ‘hyperlactation’ may be considered as an adjunct. Yet there is no workable definition of ‘hyperlactation’, since the term requires comparison with a state of ‘normal’ milk production. Normal breast milk volumes are highly variable between individuals, ranging from 478 to 1356 mls over a 24 h period in women who are exclusively and successfully breastfeeding [45]. Also, a woman successfully exclusively breastfeeding twins, generating milk volumes of two litres over a 24 h period, is not in a state of ‘hyperlactation’. The term ‘production mismatch’ more accurately identifies the contextual nature of milk production.

Clinical Protocol #36 doesn’t offer explicit pathophysiological mechanisms by which ‘hyperlactation’ causes stromal oedema and hyperaemia. Subsequently, proponents have argued that ‘hyperlactation’ causes lactose to leak through intra-lactocyte tight junctions to inhibit milk secretion and to penetrate the stromal space, increasing interstitial fluid volume [46, 47]. Problems such mechanism of penetration of the basement membrane are not addressed. The studies cited to support this ‘hyperlactation’/lactose hypothesis of breast inflammation more plausibly corroborate the mechanobiological model of breast inflammation [4].

Infant lactose overload or maternal recurrent breast inflammation as a result of production mismatch, with production exceeding the infant’s caloric needs, may occur more often in sociocultural contexts where mechanical milk removal appears to occur more commonly, for example, in the United States [48, 49].

### Sources not reliably attributed

Clinical Protocol #36 does not comply with scientific standards for image description and source attribution, which may mislead clinicians. For example, scientific guidelines require authors to specify preparation, type of equipment used, and resolutions of an image at acquisition and downstream after processing [50].

Details such as source, anatomic site specifics, preparation, type of organism, and magnification for the three images in Fig. 4 entitled “Human milk microbiota”, “Healthy mammary gland” and “Mastitis” are missing. Two images from Fig. 4 are repeated in Fig. 17, again with explanatory details missing, other than the labels “Mammary duct – NO mastitis” and “Mammary duct - Mastitis *S. epidermidis* biofilm” [1]. In personal communication, Professor Juan Rodriguez (22 September 2021) explains that the mastitis image is of tissue biopsied from an area of mastitis in a lactating human breast. The extracted tissue had fixative applied before being photographed under electron microscopy at 5000x magnification.

Finding *Staphylococcus epidermis* biofilm in lactiferous ducts after biopsy and fixative does not corroborate the hypothesis that lactiferous ducts are lined with physiological biofilm in vivo, nor that pathological *Staphylococcus epidermis* biofilm in lactiferous ducts causes breast inflammation. Moreover, a 2022 Australian nested case-controlled study examined the breast milk of 20 women with mastitis and 16 women without mastitis, and did not find any clear association between *Staphylococcus epidermidis* and mastitis [51].

### Strength of recommendation taxonomy (SORT) not reliably applied

Clinical Protocol #36 provides an analysis of existing research using the 2004 Strength of Recommendation Taxonomy (SORT) [52]. SORT recommendations are intended to be based “on a body of evidence (typically more than one study)” [48, p. 549], compiled from comprehensive review of all existing evaluations of that specific intervention [52].

It is not clear that comprehensive review of all existing evaluations has been undertaken in the preparation of Clinical Protocol #36, since relevant studies are omitted (Table 1). Also, studies which are highly heterogenous and which apply a wide range of measures have been grouped together to create recommendations. Clinical Protocol #36 acknowledges that many of its recommendations derive from Level C evidence which includes consensus, usual practice and/or opinion. However, high levels of subjectivity in Clinical Protocol #36’s application of SORT mean that its rating of evidence is unhelpful for clinicians.

In one example, Clinical Protocol #36 recommends use of probiotics for lactation-related breast inflammation on the basis of SORT Level of Evidence 1–2 and Strength of recommendation B. To reach this SORT recommendation, the authors group together and analyse a scoping review by Barker et al. 2020, a Cochrane review by Crepinsek et al. 2020, a narrative analysis by Amir et al. 2016, and a narrative review of bovine and human milk microbiomes by Oikonomou et al. 2020 [29, 39, 53, 54]. Barker et al. does not support Clinical Protocol #36’s SORT recommendation for reasons detailed in Table 1 [49]. Crepinsek et al. investigated the use of probiotics as *prevention* of mastitis after childbirth (finding very low certainty of evidence) not as *intervention* for breast inflammation. Amir et al. note that probiotics are vigorously marketed for treatment of mastitis despite lack of reliable evidence demonstrating efficacy. Oikonomou et al. briefly analyse both bovine and human evidence under a subtitle “Can we manipulate the milk microbiota in order to improve mammary gland or offspring health?”, citing two human studies and drawing no conclusions [39, p. 10].

In a second example, Clinical Protocol #36 recommends avoidance of nipple shields on the basis of SORT Level of evidence 3. Strength of recommendation C. To reach this conclusion, ABM Clinical Protocol #36 considers just one 2010 study [19], and doesn’t include more recent studies and a systematic review on this topic, further discussed in Table 1.

### Reliance upon single case reports

Case reports, such as in Figs. 10 and 11, are unable to address multiple potential confounding factors. For this reason, they may mislead clinicians and are best avoided in clinical guidelines [1].

### Clinical Protocol #36 makes recommendations which may risk worsened outcomes for breastfeeding pairs and their families

In summary, Clinical Protocol #36 recommends:

- Interventions which have been investigated but lack convincing evidence of efficacy;
- Interventions which have been subject to very few evaluations and which also lack pathophysiological rationale; and
- Avoidance of an intervention which has demonstrated positive effects in management of breastfeeding problems.

As a result, some or many of Clinical Protocol #3’s recommendations may have no benefit or may even worsen outcomes for breastfeeding pairs and their families. Baeza et al. draw a similar conclusion [2].

### Clinical protocol #36 recommends unnecessary interventions which increase risk of unintended outcomes

Recommendations by Clinical Protocol #36 which lack an evidence-base or credible theoretical frame, such as ‘lymphatic drainage’, therapeutic ultrasound, and lecithin, are discussed in Table 1. These recommendations may appear benign. However, unnecessary interventions increase risk of unintended outcomes, exacerbate patient anxiety and disempowerment, increase financial burden, and may be accessible only by affluent patients within advanced economies [55–59].

### Clinical Protocol #36 recommends to not increase or to reduce milk removal when the breast becomes inflamed, which may risk worsened outcomes for breastfeeding pairs

Clinical Protocol #36 states that “overfeeding from the affected breast …. is a major risk factor for worsening tissue edema and inflammation” [1, p. 367]. By foregrounding the theoretical model of ‘hyperlactation’ as a key aetiological factor for breast inflammation without clear criteria for diagnosing ‘hyperlactation’, Clinical Protocol #36 derives the recommendation that milk removal should not be increased or be reduced when breast inflammation presents. This recommendation occurs in six places.

1. Under the heading Key Information: Pathophysiology of Mastitis Spectrum Conditions: “Reducing milk removal may transiently increase pain and erythema …; however, it ultimately prevents future episodes” [1, p. 361].
2. Under the subheading Ductal narrowing (e.g., “plugging”): “Patients may feel relief of a “plug” with breastfeeding because this decreases alveolar distension. However, repeated feeding in an attempt to relieve the “plug” will suppress FIL, increase milk production, and ultimately exacerbate inflammation and ductal narrowing” [1, p. 363].
3. Figure 10: “Patient with early inflammatory mastitis … was treated with ice, ibuprofen, acetaminophen, and feeding first off the left, less congested breast first to avoid overstimulation of the affected right breast” [1, p. 365].
4. Figure 11: “Bacterial mastitis that progressed from early inflammation in the inner quadrant to all quadrants being affected. This patient also pumped and continually fed the infant …. in an attempt to prevent ‘milk stasis’. This approach resulted in worsened ductal inflammation and bacterial overgrowth as well as milk obstruction” [1, p. 366].
5. Under the heading “Spectrum-wide recommendations c. Feed the infant on demand, and do not aim to “empty” the breasts”: “In some instances, in which the retroareolar region is so edematous and inflamed that no milk is expressible by infant breastfeeding or hand expression, the mother should not continue to attempt feeding from the affected breast during the acute phase” [1, p. 367].
6. Figure 19: “Ice and decreased removal of breast milk reduce ductal narrowing and breast swelling” [1, p. 370].

The belief that an infant can overfeed from or overstimulate the breast contradicts the evolutionary biology model which underpins the Neuroprotective Developmental Care concept of frequent and flexible breastfeeds, necessary for adequate milk production and infant weight gain [5]. From the mechanobiological model’s perspective, frequent milk removal is integral to the downregulation of breast inflammation, because the ductal dilations of milk ejection reduce intra-alveolar pressures and counter stromal pressures [5]. A true case of production mismatch, with supply exceeding the infant’s caloric needs, is identified at presentation but addressed once breast inflammation has resolved. No matter how swollen and inflamed the areolae are, clinical support continues to facilitate positional stability of the infant and milk removal.

Breast inflammation is already associated with subsequent low milk production [60]. Clinical Protocol #36’s key recommendation of not increasing or reducing milk removal has two effects predicted by the mechanobiological model, both of which risk worsened inflammatory cascades:

1. Perpetuation of excessive intra-luminal pressures;
2. Limited episodes of ductal dilations.

Worsened inflammatory cascades are predicted to worsen the clinical presentation of breast inflammation, and to result in a greater decrease in milk production post-resolution.

### Clinical Protocol #36 introduces terms or diagnoses which are poorly defined and potentially confusing or misleading

New diagnoses for clinical presentations in breastfeeding pairs should be introduced with great caution. Overdiagnosis and overtreatment are escalating international trends, including in breastfeeding women and their babies, driving unnecessary costs for families and health systems and risking unintended outcomes [55–59]. For further discussion of unnecessary or poorly defined terms or diagnoses in Clinical Protocol #36, see Table 2.

**Table 2:** Unnecessary and poorly defined diagnoses in Clinical Protocol #36 may increase the risk of antibiotic overtreatment

Quote from Clinical Protocol #36 [1]	Analysis
“Inflammatory mastitis presents as an increasingly erythematous, edematous, and painful region of the breast with systemic signs and symptoms such as fever, chills, and tachycardia” [1, p. 363]	Mastitis means ‘inflammation of the breast’. • Because all mastitis is inflammatory, the tautology ‘inflammatory mastitis’ is not a useful term or diagnosis. • More severe presentations on the spectrum of mastitis are associated with systemic signs and symptoms [5], not necessarily because of bacterial overgrowth [44].
“Bacterial mastitis represents a progression from ductal narrowing and inflammatory mastitis to an entity necessitating antibiotics or probiotics to resolve … Bacterial mastitis presents as cellulitis (worsening erythema and induration) in a specific region of the breast that may spread to different quadrants … An evaluation by a medical professional should be performed if there are persistent systemic symptoms (> 24 hours) such as fever and tachycardia. In the absence of systemic signs and symptoms, diagnosis should be considered if the breast is not responding to conservative measures” [1, p. 363] “e. Reserve antibiotics for bacterial mastitis” [1, p. 370]	Signs and symptoms which indicate a progression from ‘inflammatory mastitis’ to ‘bacterial mastitis’ are not able to be defined. • Recommendation for a medical professional assessment after 24 h of systemic signs implies that after 24 h ‘inflammatory mastitis’ may have become ‘bacterial mastitis’, requiring treatment with antibiotics or probiotics (see **Table 1** for discussion re probiotic efficacy). • Human milk and breast stromal bacteria interact with and are altered by all presentations of breast inflammation [4]. • Cellulitis is a bacterial skin infection; mastitis is an inflammatory condition of the breast stroma, associated with secondary inflammatory changes in the skin. • Fevers are not linked to abscess formation; even with fever, most breast inflammations resolve with conservative measures, including fit and hold intervention and increased frequency of feeds [5] [60]. • Persistent signs and symptoms at the most severe end of the spectrum of breast inflammation over the passage of multiple days may require antibiotics.
“Phlegmon should be suspected with a history of mastitis that worsens into a firm, mass-like area without fluctuance. … Acute bacterial mastitis … can progress to phlegmon. Lactational phlegmon may require extended antibiotics for complete resolution, but cases should be considered individually” [1, p. 373].	This use of the term phlegmon risks unnecessary imaging and antibiotic use [5]. • Term ‘phlegmon’ is poorly defined and used inconsistently in medical practice [61], referring variously to a localised area of soft connective tissue inflammation; an inflammatory mass; diffuse, spreading inflammation; or cellulitis [62]. • An abscess is a collection of pus walled-off by granulation tissue, distinct from phlegmon. • Lactational phlegmon cannot be diagnosed by a specific set of presenting signs and symptoms. It can’t be both a tender, erythematous and non-fluctuant mass on the spectrum of breast inflammation presentations, and yet also a distinct clinical entity [1, 63]. Radiologists diagnose phlegmon by subjective criteria when imaging a lactation-related lump to exclude abscess. • Although the finding of a phlegmon on imaging indicates greater inflammatory severity due to discernible amounts of interstitial fluid, there is no rationale for the assertion that phlegmonmay be bacterial, requiring an extended course of antibiotics [63]. • Close clinical monitoring is required. No follow up imaging necessary if presenting signs and symptoms resolving [5]. • Antibiotic use indicated if signs and symptoms of breast inflammation worsening as multiple days pass rather than resolving, or because imaging has identified an abscess [5].
“Subacute mastitis occurs when ductal lumens become narrowed by bacterial biofilms in the setting of chronic mammary dysbiosis” [1, p. 365]	This use of the term subacute mastitis with its associations of biofilm and mammary dysbiosis increases risk of unnecessary antibiotic use. • Subacute mastitis unable to be defined by presenting signs and symptoms. • See elsewhere for analysis of studies which claim to define subacute mastitis [5].
“A galactocele develops when ductal narrowing obstructs the flow of milk to the extent that a significant volume of obstructed milk collects in a cyst-like cavity” [1, p. 365] “Galactoceles, which can result from unresolved hyperlactation, can become infected” [1, p. 360] “An infected galactocele requires drainage as well as antibiotics” [1, p. 373]	This pathophysiological theory of galactocoele development doesn’t consider alveolar rupture, associated apoptosis, and resultant tissue destruction which occur in subclinical inflammatory development of galactocoeles [5]. • Pathophysiological mechanism by which galactocoeles are hypothesised to result from ‘hyperlactation’ not described. • Term ‘infected galactocoele’ is redundant because galactocoele which becomes infected has become an abscess.

#### Example A. Erroneous use of the diagnosis ‘lymphedema’

Clinical Protocol #36 confuses the temporary increase in breast stromal interstitial fluid associated with inflammation with the medical condition of lymphoedema. The diagnosis of lymphoedema is only relevant to the lactating breast in the exceptional case of a genuine primary or secondary lymphoedema co-morbidity.

Secondary or acquired lymphoedema is a chronic and progressive disease. It occurs subsequent to destruction of normal lymphatic vasculature by systemic disease, trauma, or surgery. Secondary lymphoedema often results in fibrosis [64]. Although the most common cause of secondary or acquired lymphoedema world-wide is filariasis, in advanced economies the most common cause is surgical excision or irradiation of lymph nodes due to breast cancer treatment, predominantly affecting the upper limbs and occasionally the breast. The phenotypes of primary lymphoedema are rare, mostly genetic, and also often progressively fibrotic [65].

Clinical Protocol #36 states in Fig. 5: “Day 5 postpartum breast engorgement showing edematous nipple areolar complex and dependent lymphedema with overlying erythema” [1, p. 363]. This patient may have some increased interstitial fluid and stromal tension, but her nipple areolar complex swelling is most likely from high intraluminal pressures in both her alveoli and lactiferous ducts [4]. Lactiferous ducts become dilated and tense when the volume of milk produced exceeds milk removed. Most glandular tissue is subareolar, in a 3 cm radius from the base of nipple [33].

Similarly, in Fig. 21 entitled “Technique of lymphatic drainage”, Clinical Protocol #36 states that ‘lymphatic drainage’ “reduces swelling by assisting movement of lymph fluid, decreasing edema, softening fibrosis” [1, p. 371]. However, an inflamed lactating breast is not fibrotic.

Clinical Protocol #36 states: “Lactating breasts … require support to avoid dependent lymphedema” [1, p. 368]. In addition to inappropriate use of the term “dependent lymphedema”, the recommendation to “wear an appropriately fitting supportive bra” to prevent or manage mastitis may illustrate US-centrism in the development of Clinical Protocol #36 [1, p. 368], relevant because of higher levels of unhealthy body weights in the US. Such advice may have a role for a small subset of lactating women but is not, from an evolutionary and cross-cultural perspective, relevant preventative advice. External pressure from tight-fitting garments may occlude ducts and increase the risk of inflammation. Bras are a sociocultural innovation which predispose some women to breast inflammation due to pressure effects, no matter how well fitted, and require careful management when inflammation emerges [5].

#### Example B. Use of the poorly defined terms or diagnoses of inflammatory mastitis, bacterial mastitis, phlegmon, subacute mastitis and infected galactocoele

Use of the poorly defined terms or diagnoses of ‘inflammatory mastitis’, ‘bacterial mastitis’, ‘phlegmon’, ‘subacute mastitis’, and ‘infected galactocele’ in Clinical Protocol #36 risks perpetuation of unnecessary antibiotic use, in an era when the World Health Organization calls urgently for antimicrobial stewardship due to the potentially catastrophic and global consequences of antimicrobial resistances [66, 67]. These poorly defined terms or diagnoses are further discussed in Table 2 [5].

## Conclusion

As Baeza et al. 2022 have discussed [2], Clinical Protocol #36 raises important questions about the Academy of Breastfeeding Medicine’s processes for developing new clinical protocols. In response to Baeza et al’s critique, Mitchell et al. state (p. 972):

“Based on the concerns raised, we performed a detailed review of each of the comments and the relevant citations, and maintain the recommendations in the protocol are an accurate representation of the literature (at the time of publication acknowledging the scientific limitations in some areas discussed in this protocol). This is in agreement with the conclusion reached through the rigorous process of review for all protocol development, being subjected to an extensive ABM committee and Board review. ABM stands behind the protocol as written and published.” [68].

Since it is clear that recommendations in Clinical Protocol #36 do not accurately represent all relevant research literature available at the time of publication, it is possible that the Academy of Breastfeeding Medicine, its members, and breastfeeding families would benefit from an independent review of the Academy of Breastfeeding Medicine’s processes for developing Clinical Protocols.

Moreover, in light of escalating international trends to overdiagnosis and overtreatment, which benefit industry and powerful market forces, this independent review could also consider the Academy of Breastfeeding Medicine’s mechanisms for mitigating against overdiagnosis and overtreatment in all facets of membership and public engagement. Reviewing these mechanisms could allow the Academy to strengthen its leadership role not just for breastfeeding families and the health professionals who support them in advanced economies, but for breastfeeding families and health professionals who support them globally, for the sake of environmental and health system sustainability.

This Commentary aims to promote ongoing respectful debate amongst clinicians and researchers within the Academy of Breastfeeding Medicine and more broadly, confident that we share a fundamental commitment to promote breastfeeding and support the well-being of lactating women, their infants, and their families.

## Data Availability

Not applicable.
